# The integration of behavioral health consultants within a state-wide psychiatry consultation program: a community case study

**DOI:** 10.3389/fpsyt.2023.1187927

**Published:** 2023-08-17

**Authors:** Elizabeth Tengelitsch, Erin Hughes-Krieger, Dayna LePlatte, Samantha Shaw-Johnston, Sheila Marcus

**Affiliations:** Department of Psychiatry, University of Michigan, Ann Arbor, MI, United States

**Keywords:** integrated care, behavioral health consultation, mental health consultation, collaborative care, psychiatry access program

## Abstract

This paper describes the integration and role of masters-prepared behavioral health consultants (BHCs) within a state-wide psychiatry consultation program for children, adolescents, and perinatal women. Data from the Michigan Clinical Consultation and Care (MC3) program are reviewed, with attention to the role that BHCs play in the consultation process, integrated care, screening, and their dual roles with Community Mental Health Services Programs (CMHSPs) in Michigan. Approximately 82% of MC3 services are provided by the BHCs and involve resources or integrated care. BHCs play a role in managing provider consultations and connecting patients and providers to resources in the MC3 program.

## Introduction

Mental health conditions are common in childhood, adolescence, and the perinatal period. Half of all adolescents will have a diagnosable mental health condition before age 18 (1), and 40–80% of adolescents do not receive treatment (2), with racial and ethnic minority children and youth less likely to receive care than white children and youth (3). The perinatal period is intimately related to child mental health, as many mothers who experience depression or trauma during this period have children with insecure or disorganized attachment relationships, which affects children’s development and later life trajectory (4). Additionally, the prevalence of perinatal mental health conditions has increased over time (5) and the cost of an untreated perinatal mental health condition is estimated at $32,000 per mother–child dyad from pregnancy until the child is 5 years old (6).

Access to psychiatric care is a significant treatment barrier for children, adolescents, and perinatal women, with a shortage of child, adolescent, and perinatal psychiatrists across the country (7, 8), and pediatric primary care providers reporting difficulty finding local child and adolescent psychiatrists (9). This deficit was highlighted during the COVID pandemic and resulted in the AACAP, American Academy of Pediatrics (AAP), and the American Hospital Association issuing a statement outlining “The National Emergency in Children’s Mental Health Care” (10). To mitigate these circumstances, many states have established regional or state-wide psychiatry consultation programs that provide phone-based, telepsychiatry, and integrated consultation services to primary care and obstetric clinicians within their states. These programs are linked through the National Network of Child and Adolescent Psychiatric Access Programs (NNCPAP) for children and youth and Lifeline4Moms for perinatal women. Primary care providers report high levels of satisfaction and higher levels of confidence in treating patients with mental health conditions because of these consultation programs (11, 12).

Previous publications about the Michigan Clinical Consultation & Care (MC3) program^1^ described the establishment of a child psychiatry access program (13), how a psychiatry access program can partner with primary care for preschool age children (14), and primary care provider perceptions of efficacy and improved competence after consulting with a psychiatry access program (11). This paper contributes to the literature by describing the network and specific role of the behavioral health consultants (BHCs) in MC3, which serves primary care providers who treat children, youth, and perinatal women across Michigan. Many programs have siloed perinatal and pediatric programs (15), whereas MC3 integrates pediatric and perinatal because of the interrelatedness between maternal and child mental health.

MC3 is funded through the state of Michigan via state, Medicaid, and Health Resources and Services Administration grants and provides psychiatric and behavioral health consultations to primary care providers. The MC3 team consists of child and perinatal psychiatrists who are located at the University of Michigan as well as 12 full and part-time BHCs who are geographically dispersed across the state. The urban areas of Michigan are in the southern area of the state, and more than 90% of the state is classified as rural (16). Commuting distances between major hospital hubs and specialists in the southeast metropolitan Detroit region and the most rural areas make specialty care inaccessible to rural populations.

The MC3 network of BHCs bridge the gap between the MC3 child and perinatal psychiatrists and the unique needs of each region. BHCs are masters-prepared mental health professionals who are employed by the local Community Mental Health Services Programs (CMHSPs) but funded through the MC3 program. This arrangement allows the BHCs to connect with local resource information available to regional CMHSPs and disseminate this information to providers enrolled in MC3. MC3 BHCs work in the community and located in diverse regions including rural, suburban, and urban populations. The BHCs understand their regions’ needs, local mental health attitudes, trauma prevalence, and other issues such as LGBTQ+ community acceptance and the how the local culture influences how to approach families about guns. Additionally, BHCs work with community stakeholders such as Community Mental Health leadership, health system leadership and clinic leadership, regional perinatal collaboratives, local school districts, and local health departments to discuss regional issues and barriers, additional services available, and other topics that affect perinatal women, children, and families. The regional connection also facilitates the BHC’s ability to navigate local referrals for eligible children/youth and the CMHSP system.

## Context and program model

### Roles and responsibilities of behavioral health consultants

**Figure 1:**
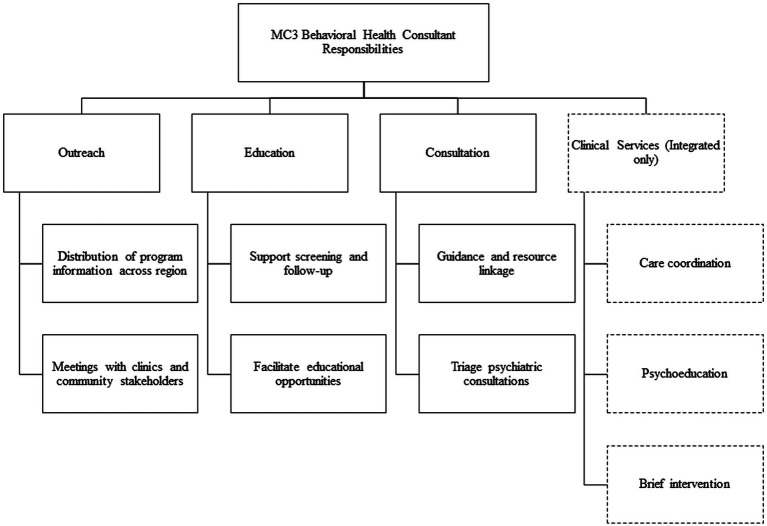
Behavioral health consultant responsibilities.

The BHC has numerous responsibilities within the MC3 program (see Figure 1). Their qualifications include a master’s degree and licensure in social work, psychology, or professional counseling, as well as knowledge of the regional Community Mental Health Services Program (CMHSP), their provider network, community resources, and familiarity with the Michigan Mental Health Code (17). They are knowledgeable about regional school-based programs and the Department of Health and Human Services, including child protection services, foster care services for youth, as well as community and home-based services available to youth and perinatal women through CMHSP.

In addition, BHCs are skilled in brief intervention modalities such as motivational interviewing and have backgrounds in evidence-based treatment such as cognitive and dialectical behavioral therapies, interpersonal therapy, Child–Parent Psychotherapy, and working with high-risk dyads. These skills are essential for BHCs who are integrated in primary care practices and providing brief, therapeutic interventions. Desired qualifications also include the skill set required for provider outreach and engagement, such as strong communication skills, the ability to speak confidently with community administrators and physicians, and willingness to travel through the region to present program information.

The BHCs are recruited, hired, and supervised by the local CMHSP. MC3 administration provides orientation and training for the BHCs on their MC3 roles and responsibilities and ongoing support and guidance to ensure the BHCs are following standard operating procedures, along with troubleshooting day-to-day questions or concerns. MC3 administration meets with the BHCs individually and as a group monthly to discuss provider engagement, community needs, and program questions. These meetings also provide the BHCs with support in the difficult work they are doing within their communities, addressing self-care before transitioning to discussion around shared work responsibilities and program updates. With COVID, additional opportunities were created for BHCs to connect with the MC3 administrative team and each other through daily Zoom yoga sessions, meeting with the consulting psychiatrists as needed. MC3 also plans and holds an annual BHC 2-day meeting for all BHCs where training and opportunities for networking and team building occur.

The MC3 BHC has a unique role as an employee of the local CMHSP. BHCs are familiar with the range of services offered within the CMHSP and the eligibility criteria for services. The BHC can more easily triage psychiatric consultations and advocate for children, youth, and perinatal women in need of those services. When individuals receiving care at CMHSP are stable and medications can be managed within primary care, they are referred to primary care providers (PCPs) for ongoing clinical management. BHCs work closely with PCPs during this transition to provide support and facilitate MC3 consultation to providers treating these individuals. The role of the MC3 BHC is a liaison and a bridge between CMHSP/specialty behavioral health services and primary care. Improved communication and increased understanding benefits perinatal women, children/youth and their families who are trying to navigate a new service system. Regional BHCs also help reduce disparities by ensuring that a BHC is available, regardless of population concentration or geographic location.

The MC3 BHC coordinates regional outreach to eligible providers to engage them in the service and ensure utilization of the program. Eligible providers include physicians, obstetricians, certified nurse midwives, nurse practitioners, physician assistants, and physician residents in primary care settings. BHCs begin by building a directory of health systems, clinics, and providers eligible to participate in the program within their designated region. Next, BHCs identify contacts in each health system and clinic and initiate contact through phone calls, emails, and letters to invite providers to enroll or to schedule an informational or enrollment meeting. In these meetings, BHCs share the details about program services and how providers can participate in all levels of consultation and education. The BHCs also utilize meetings to gather additional regional information by inviting an open dialog with providers to discuss their needs and how MC3 can help with the population they serve. Once providers have enrolled, the BHC maintains regular contact with the clinics to sustain relationships and ensure successful engagement in program services. Providers are encouraged, but not required, to complete a survey upon enrollment that asks about their patients’ access to services and the provider’s comfort and confidence in diagnosing and managing common mental health problems in primary care. The BHCs send providers a welcome email and a packet is mailed out with program information, magnets with contact information, and psychopharmacology reference cards. The BHCs reach out to providers annually to remind them of the program services and ask for an opportunity to meet with practices to review program updates.

In select clinics, BHCs facilitate targeted education on topics of interest such as developmental or behavioral health screening or suicide safety planning, and community resources (i.e., crisis resources). BHCs assist the psychiatrists in providing guidance to PCPs regarding additional screening and measurement-based care. They make recommendations for which measures to use and provide copies of these measures during consultations and informational meetings. BHCs also facilitate educational opportunities and case conferences on a wide range of topics. They facilitate workflow meetings to assist practices in integrating consultation services and/or screening into their clinic. Additionally, the BHCs coordinated psychiatrist-led Provider Cafes during the COVID-19 pandemic. These cafés provided a virtual group venue for reflective consultation in which providers received support for burn-out and an opportunity to reflect on the challenges faced during the pandemic including compassion fatigue and stress management.

Once eligible clinics and providers are engaged, the BHC provides linkage between PCPs and MC3 psychiatrists. PCPs request same-day psychiatry consultation by contacting the regional BHC by phone or through online submission. The state is divided into 7 regions and each region has a toll-free number that is covered by regional BHCs. Regions with more than one BHC rotate phone coverage, with each BHC to cover the regional phone 1–3 days a week. The BHC collects de-identified patient information, the presenting problem, address questions within the BHC’s scope, and facilitate the consultations between the psychiatrist and PCP, to occur the same day as the request, or the next day if the call is received toward the end of the business day. Providers also have the option to schedule the consult on a day and time that works best for them. After the consultation, the BHC summarizes the psychiatrist’s recommendations and provides related community resources or patient handouts to the provider within 24 h of the consultation. When a full telepsychiatric evaluation is recommended after a consultation, the MC3 program facilitates this by having the BHC coordinate the referral and make direct contact with the patient before and after the evaluation to assist in coordination and linkage to community resources. Typically, the 90-min evaluation is scheduled within 4–6 weeks of the initial MC3 consultation and may result in additional referrals for further evaluation as warranted, such as neuropsychological or Autism Spectrum Disorder testing.

Each BHC is responsible for building and maintaining a directory of local and validated resources and services to provide to patients and providers. They identify resource information within the identified region for children, adolescents, and perinatal women based on recommendations by the MC3 consulting psychiatrist. Resources may include referrals for psychotherapy, school-based resources, or other community-based services. Referrals provided by BHCs are tailored to individual patients and families, with modality, geographic location, and insurance coverage taken into consideration. Such targeted referrals assist patients and families in navigating the mental health system and securing needed services.

Finally, some BHCs are integrated in designated primary care sites and provide direct integrated services to patients. A site is selected to receive an integrated BHC based on several different factors: (1) higher Medicaid population, (2) desire to improve behavioral health services at the site and providers’ willingness to treat behavioral health, (3) lack of existing site behavioral health supports, (4) motivation to work with MC3 to integrate the BHC into the site. The integrated BHCs offer brief mental health assessments to assist with referrals to appropriate community providers. They also offer brief interventions to patients and their families that may include anticipatory guidance, psychoeducation, motivational interviewing, information on services available to address identified concerns, or other approaches as appropriate for age and disposition. Referrals for brief intervention are often warm handoffs after a PCP appointment or, if the BHC is not available at the appointment, the clinic makes a referral to the BHC who then calls the family to follow-up. Brief interventions are typically between 1 and 5 visits by phone, video, or in-person at the PCP office, and 20–30 min in length, using a shared medical record and scheduling system. Patients with mild to moderate behavioral health symptoms who receive these brief interventions may not need to be referred to specialty services, whereas more severe patients can be bridged by the BHC until they are able to engage in specialty care. Integrated BHCs monitor patient progress and symptom improvement through follow-up calls using screening tools such as the PHQ-9 and GAD-7 for those patients seen for brief interventions with depression and/or anxiety. The Columbia-Suicide Severity Rating Scale (C-SSRS), Ask Suicide-Screening Questions (ASQ) and Stanley-Brown Safety Plan are used with patient who endorse thoughts of suicide. The BHCs have all participated in the Counseling on Access to Lethal Means (CALM) course and provide consultation to clinic staff on these tools.

The BHC will often participate in individual and team meetings with primary care providers and office staff to discuss patients who are served in the practice. BHCs follow up with the patient/family to help the providers monitor adherence to treatment, ensure linkage to resources and monitor progress toward treatment goals. In designated clinics, BHCs participate in panel reviews with the consulting psychiatrist where patients are reviewed, and recommendations are shared with the treating provider. Integrated BHCs often recommend PCP consultation with MC3 psychiatrists when needed.

Productivity targets for BHCs depend on level of integration. Integrated BHCs are expected to provide integrated services for 5 patients each day they are in a clinic, whereas on phone coverage days, integrated and non-integrated BHCs should anticipate at least 3 phone consultations. Remaining BHC time includes meetings, training, documentation, and clinic outreach, with the expectation that every clinic will receive at least one outreach attempt annually, which also includes verification of providers at each clinic.

## Program data

### Overview

Data is collected through a program administration database as well as a secure, HIPAA-compliant electronic health record system. The first author (ET) is the program analyst and compiled all data, ensuring that an anonymized, aggregated dataset was utilized for analysis. The University of Michigan IRB determined MC3 and its activities to be under quality assurance and quality improvement, and therefore MC3 is classified as not regulated.

There are 12 BHCs who serve 83 counties in Michigan, and more than 3,100 enrolled primary care providers (PCPs). PCPs are primary care providers including pediatricians, obstetricians, family medicine physicians, nurse practitioners, physician assistants, and residents. Only full-time BHCs are integrated in primary care offices. As of 2023, 6 of 8 full-time BHCs were integrated within 8 PCP offices, whereas the other 4 BHCs were part-time and not integrated in any office. In addition to primary care offices, the BHCs serve more than 40 school-based health centers, where clinicians provide primary care for adolescents. Since program inception, BHCs have facilitated over 1,200 informational and enrollment meetings, and 200 case conference meetings.

### Utilization data (2012–2021)

Since program inception in 2012, there have been over 38,000 service encounters for more than 15,000 patients. Service encounters consist of BHC to PCP consults, BHC triage of psychiatrist to PCP consults, psychiatrist to PCP consults, psychiatrist to patient telepsychiatry evaluations, BHC follow-up of psychiatrist to PCP consults, BHC-psychiatrist patient panel review, BHC to PCP panel reviews, and BHC to patient direct services (see Table 1).

**Table 1:** Service encounter types, 2012–2021.

Service encounter type	Definition	% (*n*)
BHC to patient direct service	BHC provides face-to-face, phone, or virtual direct service to patient.	35.3% (13,635)
BHC triage of psychiatrist consult/telepsych evaluation	BHC collects patient history and reason for call, coordinates same-day consult between psychiatrist and PCP for same-day phone consult or scheduled telepsych evaluation between psychiatrist and patient.	19.2% (7,445)
BHC follow-up of psychiatrist consult/telepsych evaluation	BHC reviews consult and provides written summary of consult and community resources.	19.2% (7,445)
Psychiatrist to PCP consult	Psychiatrist consults with PCP and makes recommendations for management of patient.	19.0% (7,362)
BHC to PCP consult	BHC triages PCP consult request, responds to any questions within the scope of expertise, and suggests local resources for referral.	6.5% (2,495)
BHC-psychiatrist patient panel review	BHC meets with psychiatrist to review all new patient referrals and all patients who are not showing progress on outcome measures.	0.3% (108)
BHC to PCP panel review	BHC reviews psychiatrist recommendations and follows up with PCP and patient.	0.3% (108)
Psychiatrist telepsych patient evaluation*	Psychiatrist evaluates patient and makes recommendations to PCP for ongoing care.	0.2% (83)

*During a psychiatrist to PCP consult, additional assessment may be warranted in the form of a psychiatrist to patient telepsychiatry evaluation. The role of the BHC is to initiate the referral for this evaluation. These evaluations are separate from the MC3 consultation program and billed through insurance.

**Figure 2:**
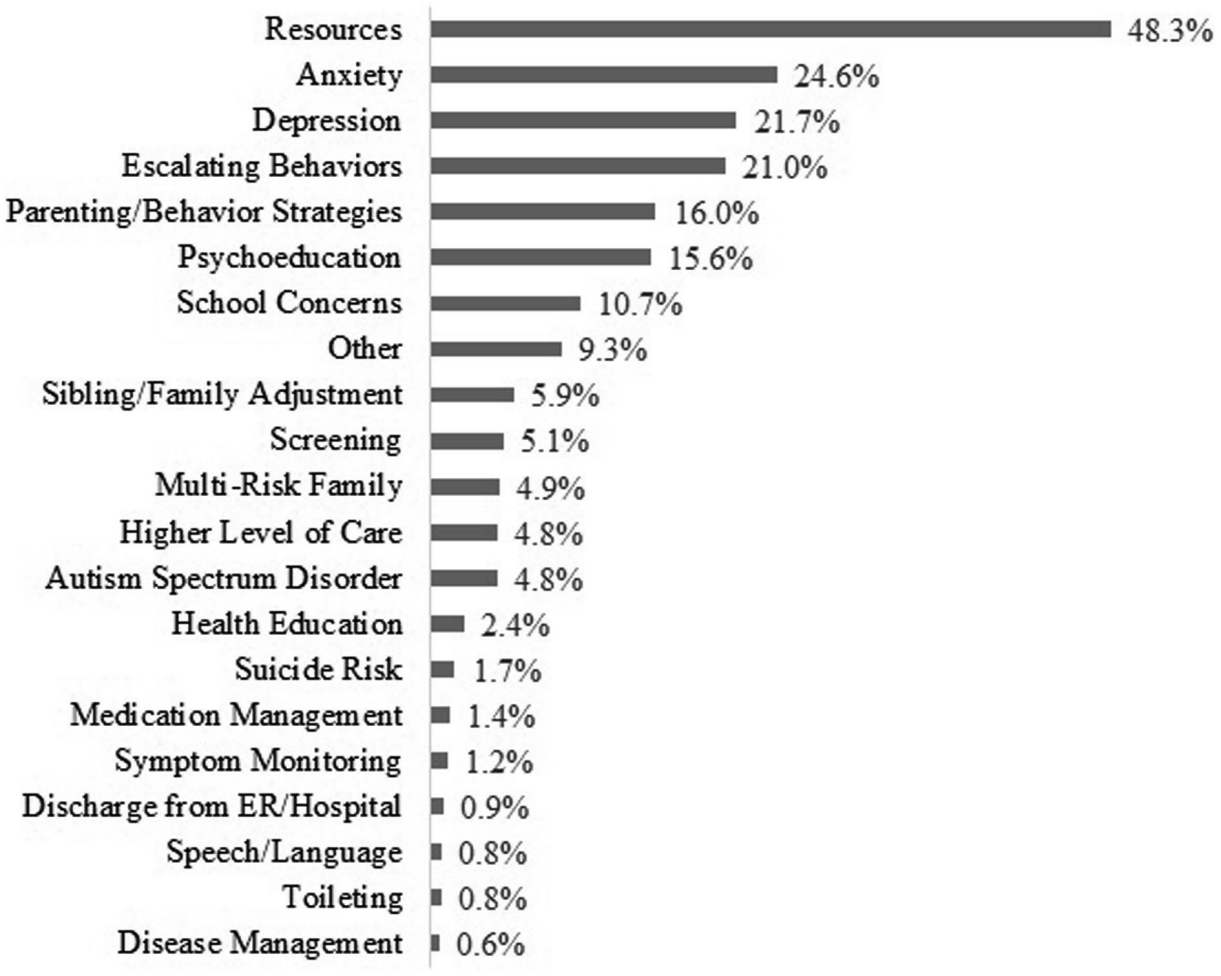
Referral reason for BHC direct services.

**Figure 3:**
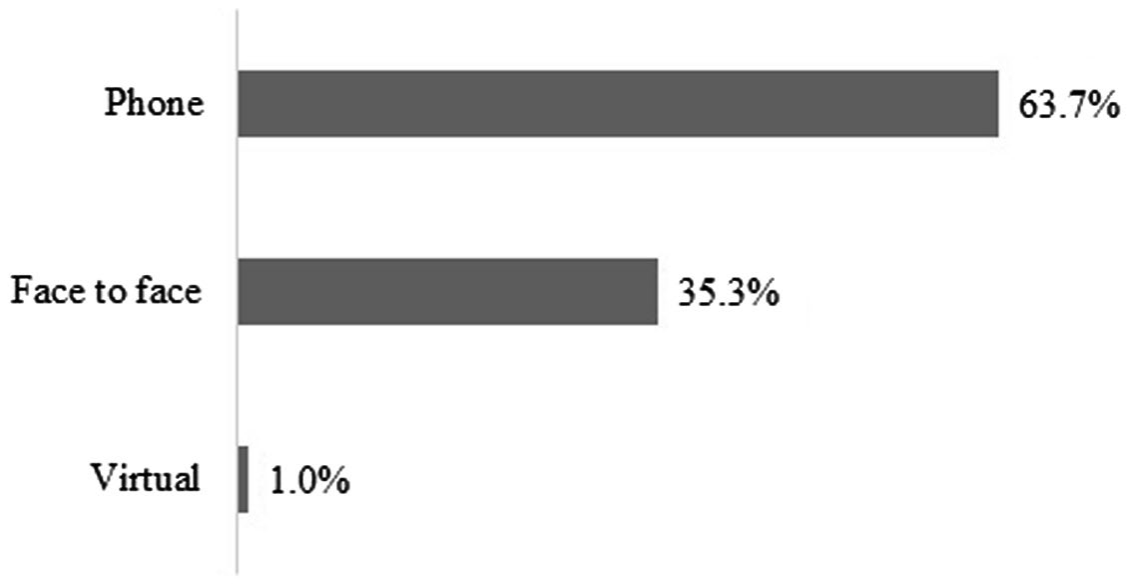
BHC direct service by modality.

BHCs were involved in 80.8% of all service encounters, with 41.8% performed exclusively by BHCs (see Table 1). BHC-exclusive service encounters consist of 2,495 phone-based resource consults to PCPs and 13,635 BHC to patient direct services (see Figure 2 for service modality). PCPs refer patients for any number of reasons, and these may be for specific services (such as resources or psychoeducation) and/or the specific concern (depression, ADHD, etc.). There are often multiple reasons for referral (see Figure 3), with resources, anxiety, and depression as the most common referral reasons, with some rank order differences between pediatric and perinatal (see Table 2). Pediatric resources provided by BHCs may include information on a wide variety of topics, from handouts on toileting and relaxation techniques, to information about local therapists and navigating the CMH system. Perinatal resources are most often for depression and include information and referrals for infant mental health or counseling resources in the area.

**Table 2:** Top 10 reasons for pediatric and perinatal referrals.

Pediatric	Perinatal
1. Resources	1. Depression
2. Anxiety	2. Resources
3. Escalating behaviors	3. Other
4. Depression	4. Anxiety
5. Parenting/behavior strategies	5. Screening
6. Psychoeducation	6. Parenting/behavior strategies
7. School Concerns	7. Multi-risk family
8. Other	8. Sibling/family adjustment
9. Sibling/family adjustment	9. Psychoeducation
10. Autism spectrum disorder	10. Health education

## Discussion

The BHCs have played a critical role to the success of the MC3 program in Michigan. Their role as the liaison between the psychiatrist and PCP and as a consultant to the PCP with expertise in behavioral health resources has been the backbone of the MC3 program. The regional distribution of the BHCs is key to state-wide coverage and local expertise in behavioral health resources matched with PCP preferences, payer types, and clinician availability. This timely information is helpful to local PCPs attempting to help their patients access essential resources. The BHCs are linked to regional Community Mental Health Services Programs and have knowledge of eligibility criteria for access to specialty care, which facilitates linkage to care and can support PCP management of individuals who are deemed stable enough to be stepped down from CMHSP.

An additional unique aspect of the MC3 program is that BHCs are familiar with both resources and educational materials for infants and young children and perinatal women. They are aware of the need to support pregnant and postpartum women such as those with severe depressive illnesses and history of trauma and domestic violence. The BHCs can also coordinate care needed by a pregnant mother and her infant, particularly in instances of intergenerational trauma. Linkage to resources which support relational health and foster attachment security are important for young children and caregivers because a secure attachment relationship is associated with positive health outcomes and social functioning including better capacity for intimacy, and improved academic and social functioning (18, 19).

Many consultations are for patients with severe symptoms including history of suicidal ideations and attempts, trauma, and hospitalization. BHCs provide support by offering consultation and resources to PCPs in cases where patients are discharged from the emergency department deemed ineligible for hospitalization. The role of the BHC has been highlighted during the COVID pandemic with both state-wide and national strain on behavioral health resources.

### Challenges and barriers

The COVID-19 pandemic presented substantial barriers to the MC3 program. Opportunities to engage providers in program offerings were impacted by competing demands and reprioritization of their workload. These included switching to remote care, COVID testing, vaccination, and responding to ill patients. From March 2020 to spring/summer of (10), many clinics were overwhelmed in meeting the demand of the pandemic and were unable to accommodate new workflows or convene enrollment and informational meetings to begin MC3 consultations. In addition, many of the integrated BHCs worked from home during this period, curtailing the availability of in-person or real-time integrated services due to the change in clinic workflow with telehealth visits being the standard for many providers. However, BHCs were resourceful and continued to provide services by pivoting the MC3 framework to one that was entirely remote during this period. Although prior to 2020 many of these visits and meetings were conducted in person, during the COVID pandemic most enrollment and informational meetings occurred virtually.

As the pandemic has continued across Michigan and the entire United States, a mental health “surge after the surge” has taken hold (20). Behavioral health consultants have struggled to find psychotherapy resources with availability due to the increase in demand for therapy services and the increasing rates of depression, anxiety, and suicidality in child and adolescent patients (20, 21). Increasing rates of suicidality have led to higher numbers of emergency room visits for behavioral health conditions, in turn straining the capacity of the inpatient units to provide adequate psychiatric beds (22). This highlights the increasing importance of programs like MC3 to meet mental health needs, with.

BHCs supporting patients who require higher levels of care such as CMHSP services and assisting providers in bridging patients who are unable to be accommodated in hospitals or specialty care.

### Future directions

As the MC3 program continues to grow, there may be new opportunities for BHC involvement and integration. Recent federal grant opportunities suggest that the need for behavioral health services and psychiatry access programs will continue to grow (23). Upcoming opportunities, including partnering with other state-wide programs which provide cognitive behavioral training in schools, may afford additional resources that can be utilized by the BHCs (24). Additionally, MC3 has recently expanded consultation and services to school settings and is gathering preliminary data on providing consultation to emergency services departments as well as pediatric specialty providers. These additional areas may include hiring more BHCs as the program will reach additional populations of providers and patients, helping to ensure that patients have equitable access to resources and support from BHCs.

Access to resources and the role of insurance is another area in which BHCs may play an integral role in the future in helping patients connect with care, as lack of or type of patient insurance can be a significant barrier for patients connecting to resources.

Finally, we plan to engage in a more formal analysis of implementation outcomes of our model in the future to assess the effectiveness and sustainability of BHC services that can contribute to previous research that has examined implementation outcomes (25). Suggestions for additional research and program evaluation of psychiatry access programs, include randomized control trials to examine provider actions and patient outcomes, mixed-method studies to help understand who is best helped by these services, and cost-analyses to assess return on investment.

## Conclusion

The role of the BHC in flexibly supporting patients who await behavioral health care, particularly during the pandemic, cannot be overstated. The BHCs are a pivotal part of the MC3 program from the point of inception with enrolling and engaging physicians, to facilitating all calls and resources back to PCPs. MC3 provides a framework that should be considered in newly emerging psychiatry access programs, to ensure optimal functioning and communication of the program.

## Data availability statement

The raw data supporting the conclusions of this article will be made available by the authors, without undue reservation.

## Ethics statement

Ethical review and approval was not required for the study on human participants in accordance with the local legislation and institutional requirements. Written informed consent from the participants was not required to participate in this study in accordance with the national legislation and the institutional requirements.

## Author contributions

ET, EH-K, and SM were substantial contributors to the conception or design of the work, or the acquisition, analysis, or interpretation of data for the work. ET, EH-K, DL, and SS-J drafted the work or revised it critically for important intellectual content. SM provided the approval for publication of the content. ET and SM agreed to be accountable for all aspects of the work in ensuring that questions related to the accuracy or integrity of any part of the work are appropriately investigated and resolved. All authors contributed to the article and approved the submitted version.

## Funding

This work was funded by the Michigan Department of Health and Human Services, Medicaid Administration, Health Resources and Services Administration. This information or content and conclusions are those of the author and should not be construed as the official position or policy of, nor should any endorsements be inferred by HRSA, HHS or the U.S. Government.

## Conflict of interest

The authors declare that the research was conducted in the absence of any commercial or financial relationships that could be construed as a potential conflict of interest.

## Publisher’s note

All claims expressed in this article are solely those of the authors and do not necessarily represent those of their affiliated organizations, or those of the publisher, the editors and the reviewers. Any product that may be evaluated in this article, or claim that may be made by its manufacturer, is not guaranteed or endorsed by the publisher.

## Footnotes

^1^In 2022, the MC3 program updated its name from Michigan Child Collaborative Care to Michigan Clinical Consultation and Care to better reflect the target population and services provided.
